# Impaired desensitization of a human polymorphic α_2B_-adrenergic receptor variant enhances its sympatho-inhibitory activity in chromaffin cells

**DOI:** 10.1186/1478-811X-9-5

**Published:** 2011-02-07

**Authors:** Kristy Nguyen, Theodoros Kassimatis, Anastasios Lymperopoulos

**Affiliations:** 1 Department of Pharmaceutical Sciences, College of Pharmacy, Nova Southeastern University, Fort Lauderdale, FL 33328, USA; 2 Nephrology Department, Asklipieio General Hospital, Athens, Greece

## Abstract

**Background:**

α_2_-adrenergic receptors (ARs) mediate many cellular actions of epinephrine and norepinephrine and inhibit their secretion from adrenal chromaffin cells. Like many other G-protein coupled receptors (GPCRs), they undergo agonist-dependent phopshorylation and desensitization by GPCR Kinases (GRKs), a phenomenon recently shown to play a major role in the sympathetic overdrive that accompanies and aggravates chronic heart failure. A deletion polymorphism in the human α_2B_-AR gene (Glu301-303) causes impaired agonist-promoted receptor phosphorylation and desensitization in heterologous cell lines. Given the importance of α_2_-ARs in regulation of catecholamine secretion from chromaffin cells, we sought to investigate, in the present study, the desensitization properties and the sympatho-inhibitory activity of this variant in a chromaffin cell line. For this purpose, we expressed this variant and its wild type counterpart in the well-established chromaffin cell line PC12, and performed receptor phosphorylation and desensitization studies, as well as in vitro catecholamine secretion assays.

**Results:**

Both the agonist-induced phosphorylation and agonist-dependent desensitization of the human Glu301-303 deletion polymorphic α_2B_-AR are significantly impaired in PC12 cells, resulting in enhanced signaling to inhibition of cholinergic-induced catecholamine secretion in vitro.

**Conclusion:**

This α_2B_-AR gene polymorphism (Glu301-303 deletion) might confer better protection against conditions characterized and aggravated by sympathetic/catecholaminergic overstimulation in vivo.

## Background

Three distinct α_2_-adrenergic receptor (α_2_-AR) subtypes (α_2A_, α_2B_, α_2C_) that mediate many of the physiological actions of the catecholamines (CAs) epinephrine (Epi) and norepinephrine (NE) have been described [1]. They belong to the family of G-protein coupled or seven transmembrane-spanning receptors (GPCRs or 7TMRs) and they are linked to the inhibitory G_i/o _proteins [1]. The α_2B_-AR is critically involved in cardiovascular regulation, as its gene disruption in mice affects blood pressure responses to α_2_-adrenoceptor agonists (e.g. clonidine) [2]. Its role in the Central Nervous System (CNS) remains largely elusive. It may be important in developmental processes, since homozygous α_2B_-KO mice do not breed well [2].

Like many other GPCRs, the α_2B_-AR undergoes agonist promoted desensitization [3] initiated by the phosphorylation of the receptor in its third intracellular loop by a well-characterized family of serine/threonine kinases termed G protein-coupled receptor kinases (GRKs), the most prominent member of which is the ubiquitously expressed GRK2 [4]. The phosphorylated receptor then interacts with a certain family of proteins termed arrestins, which physically uncouple the receptor from G proteins, thus terminating receptor signaling [4].

α_2_-ARs play a very important role in autocrine feedback regulation of catecholamine secretion from the chromaffin cells of the adrenal medulla [5]. By coupling to the G_i/o _proteins, they inhibit further CA release upon their stimulation by the secreted CA, thereby participating in an autocrine negative feedback loop controlling adrenal CA secretion [5,6]. A number of recent studies by our group and others have documented the (patho)physiological importance of this α_2_-AR-mediated control of adrenal CA secretion, as deregulation of this signaling system in the adrenal chromaffin cells has been shown to underlie excessive sympathetic outflow and circulating CA levels that accompany and aggravate chronic heart failure [7-9]. More specifically, up-regulated GRK2 has been found to desensitize and down-regulate chromaffin cell α_2_-ARs extensively in HF mouse and rat adrenal glands, thus rendering these receptors nonfunctional in HF. This allows for unopposed, continuous CA secretion, which contributes to the enhanced CA levels in heart failure [7].

A common genetic variant of the α_2B_-AR subtype consisting of a deletion of three glutamic acid residues (residues 301-303) displays impaired agonist-promoted receptor phosphorylation and desensitization in various transfected cell lines [10,11], and, very recently, it was shown to be resistant to desensitization (in terms of inducing vasoconstriction) in vivo, as well [12]. Given the important role of α_2_-ARs in chromaffin cell physiology with respect to CA secretion regulation, in the present study we sought to investigate the impact of this α_2B_-AR polymorphism on desensitization and sympatho-inhibitory function of this receptor in these cells. To this end, we utilized the PC12 cell line, a rat pheochromocytoma-derived chromaffin cell line [13], which we transfected with the wild-type α_2B_-AR (WT α_2B_-AR) or the polymorphic α_2B_-AR (Del α_2B_-AR) cDNA constructs in order to express and compare head-to-head these two receptor variants in chromaffin cells. We found that desensitization of the Del α_2B_-AR is impaired also in PC12 cells, which results in enhanced inhibition of CA secretion.

## Results

### Impaired agonist-promoted phosphorylation of the Del α_2B_-AR in PC12 cells

**Figure 1:**
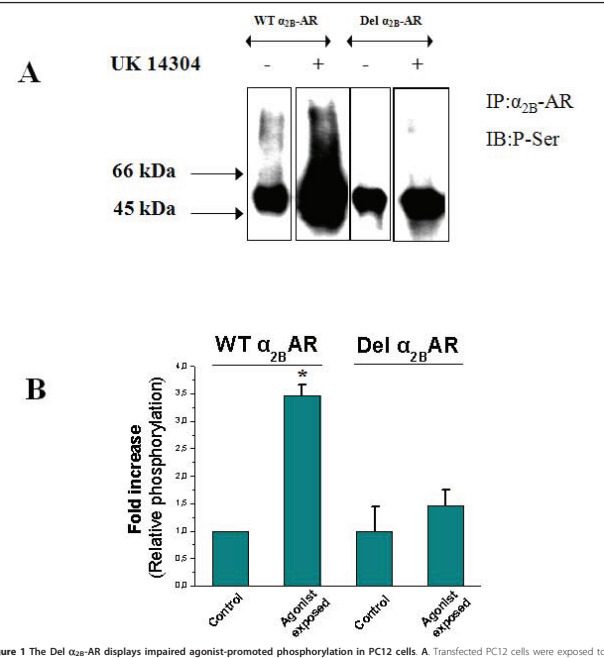
**The Del α_2B_-AR displays impaired agonist-promoted phosphorylation in PC12 cells**. **A**. Transfected PC12 cells were exposed to 10 μΜ UK 14304 for 10 min and then immunoprecipitation (IP) of the α_2B_-ARs was performed, followed by immunoblotting (IB) to measure their phosphoserine (P-Ser) content. Equal amounts of receptor were loaded on each lane, as determined from ligand binding data and protein determination by the Bradford method. Shown is a representative blot from five independent experiments. **B**. Densitometric analysis of the immunoblots of Panel A, showing agonist-induced receptor phosphorylation as fold increase of the basal phosphorylation of WT α_2B_-AR. *, p < 0.05, vs. all other conditions, n = 5.

After verifying that PC12 cells express substantial endogenous levels of both GRK2 and GRK3, more than sufficient to phosphorylate α_2_-ARs (data not shown), we performed whole cell phosphorylation studies of the two receptors in PC12 cells in response to 10 min treatments with 10 μM UK14304, an α_2_-AR-specific full agonist, or vehicle. Since GRKs are serine/threonine kinases, we used a specific polyclonal anti-phosphoserine antibody (anti-P-Ser) to examine the receptors' agonist-induced phosphorylation. As shown in Figure 1A, the Del α_2B_-AR displays indeed a dramatically decreased agonist-promoted phosphorylation compared to the WT α_2B_-AR. More specifically, WT α_2B_-AR displays ~3.5-fold increase in phosphorylation in response to agonist, whereas agonist-induced phosphorylation of the Del α_2B_-AR is virtually abolished (Figure 1B). Of note, GRK2 levels were equal between the two α_2B_-AR-expressing PC12 lines, indicating that Del α_2B_-AR transfection and overexpression did not have any effects on endogenous GRK2 levels in PC12 cells (data not shown).

### Impaired agonist-promoted desensitization of the Del α_2B_-AR in PC12 cells

**Figure 2:**
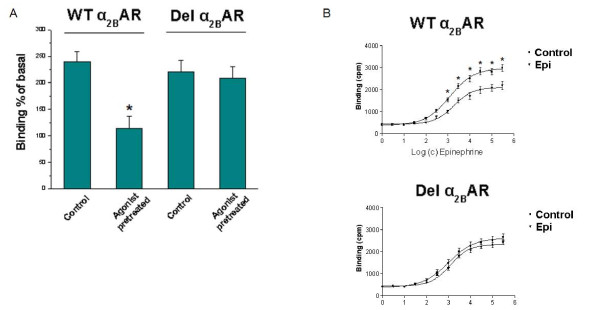
**[^35^S] GTPγS binding for the two α_2B_-ARs in PC12 cells**. **A**. PC12 cells were exposed to vehicle (Control) or 10 μM UK14304 (Agonist-pretreated) and agonist-stimulated [35S] GTPγS binding was determined. Results are expressed as % of basal binding, indicating a ~ 53% desensitization of the WT α_2B_-AR (see also Table 1). *, p < 0.05, vs. Control WT α_2B_-AR, n = 6 independent experiments. **B**. Membranes from control (Control) and 10 μM epinephrine-pretreated (Epi) cells were incubated with increasing concentrations (c) of epinephrine. Only the Epi-pretreated WT α_2B_-AR displays decreased [^35^S] GTPγS binding (i.e. desensitization) in response to Epi, whereas the Del α_2B_-AR fails to do so. *, p < 0.05, vs. Epi, n = 6 independent experiments.

We next examined the impact of this impaired phosphorylation on the functional desensitization, i.e. on the agonist-induced G-protein coupling, of the Del α_2B_-AR with the [^35^S] GTPγS binding assay. This assay is widely used to measure the G-protein coupling efficiency of GPCRs [14]. Again, we treated the PC12 cells with 10 μM UK 14304 for 30 minutes or with vehicle, and then membranes were prepared from these cells and the [^35^S] GTPγS binding assay was performed on these membranes using again 10 μM UK 14304 as agonist. As shown in Table 1 and in Figure 2A, the two non-agonist-pretreated receptors (Controls) exhibit about the same agonist-stimulated [^35^S] GTPγS binding (240 ± 13% of basal for the WT α_2B_-AR and 221 ± 15% of basal for the Del α_2B_-AR). However, the agonist-pretreated WT α_2B_-AR displays a significantly decreased agonist-stimulated [^35^S] GTPγS binding (114 ± 18% of basal), compared to the Control WT α_2B_-AR, which reflects a significant functional desensitization of this receptor (~53%). In contrast, the agonist-pretreated Del α_2B_-AR shows almost the same agonist-stimulated [^35^S] GTPγS binding (208 ± 20% of basal) as the Control Del α_2B_-AR, indicating that the Del α_2B_-AR fails to undergo desensitization in PC12 cells.

**Table 1:** Agonist-stimulated [^35^S] GTPγS binding measurements for the WT and Del α_2B_-ARs expressed in PC12 cells

	Basal	Agonist-stimulated		**Des**.
		*Control*	*UK14304*	
	Cpm/min/15 μg	%		%
WT α_2B_-AR	2983,15 ± 09,74	240,01 ± 13,02	113,74 ± 18,40^a^	53
Del α_2B_-AR	5717,39 ± 13,02^b^	221,15 ± 14,94	208,47 ± 19,93^b^	6

Agonist-stimulated [35S]GTPγS binding was determined in response to 10 μM UK14304 (agonist) in agonist-pretreated (UK14304) and in non-pretreated (Control) membranes. Des. = Desensitization.

^a ^p < 0.05 compared with control, ^b ^p < 0.05 compared with WT, n = 6 independent experiments.

In addition, GTPγS binding dose-response curves of Epi-pretreated cells revealed a significant increase (1.8-fold, p = 0.012) in the EC_50 _of Epi to stimulate GTPγS binding through the WT α_2B_-AR, whereas Epi pretreatment did not induce significant changes on the EC_50 _for the Del α_2B_-AR (Figure 2B). This result provides additional strong evidence that the Del α_2B_-AR, in contrast to its wild type counterpart which undergoes agonist-induced desensitization normally as expected, fails to desensitize in PC12 cells. This impaired desensitization of the Del α_2B_-AR comes as a consequence of its impaired agonist-induced phosphorylation in PC12 cells.

### Enhanced inhibition of catecholamine secretion by the Del α_2B_-AR in PC12 cells

**Figure 3:**
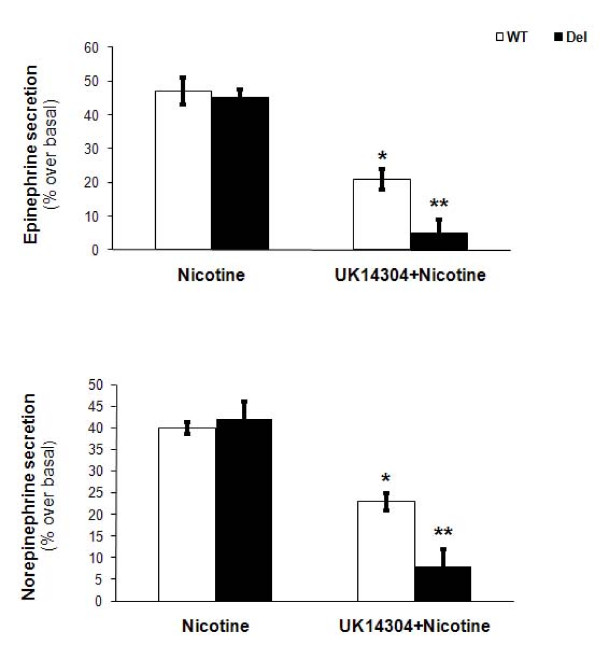
**Enhanced inhibition of nicotine-induced catecholamine secretion by the Del α_2B_-AR in PC12 cells**. *In vitro *catecholamine secretion from PC12 cells, expressing the WT α_2B_-AR (WT) or the Del α_2B_-AR (Del) and pretreated with 10 μM UK14304, in response to either 50 μM nicotine alone (Nicotine) or to 50 μM nicotine plus another challenge of 10 μM UK14304 (UK14304 + Nicotine). No differences between the two non-pretreated cell lines were observed (data not shown). *, p < 0.05, vs. WT/Nicotine, **, p < 0.05, vs. WT/UK14304 + Nicotine, n = 3 independent experiments.

Finally, we examined the functional impact of this impaired phosphorylation/desensitization of the Del α_2B_-AR in PC12 cells, and, more specifically, the impact on the ability of the receptor to inhibit CA secretion. Physiologically in chromaffin cells, CA secretion is tonically stimulated by acetylcholine acting on nicotinic cholinergic receptors endogenously expressed in chromaffin cell membranes [5]. Thus, we performed in vitro CA secretion assays in the PC12 cells using nicotine (which also stimulates nicotinic cholinergic receptors) as the stimulus for the CA secretion and UK14304 as the agonist for the α_2B_-ARs. As shown in Figure 3 nicotine induced comparable amounts of CA secretion from the two transfected and UK14304-pretreated PC12 cell lines. Of note, when no challenge with UK14304 preceded nicotine exposure, CA secretion between the two cell lines was similar (data not shown). However, when the cells were pretreated with UK14304 to induce desensitization of the α_2B_-ARs, secretion of both Epi and NE from the UK14304-pretreated PC12α_2BDel _cells was significantly reduced compared to agonist-pretreated PC12α_2BWT _cells, indicating that the impaired desensitization of the Del α_2B_-AR allows it to be a stronger inhibitor of CA secretion from PC12 cells, compared to its wild-type counterpart (Figure 3).

Notably, and because UK14304 is largely reported in the literature to be a partial agonist at the α_2B_-AR subtype, we repeated these experiments with another, full α_2B_-AR agonist, oxymetazoline, and the results were identical (data not shown). Thus, diminished desensitization of the Del α_2B_-AR mutant seems to translate into enhanced inhibitory function of the receptor on CA secretion from PC12 cells, regardless of the structure or the individual pharmacological properties of the activating agonist.

## Discussion

A polymorphic variant of the human α_2B_-adrenoceptor, which consists of a deletion of three glutamic acids (residues 301-303) in the third intracellular loop (Del α_2B_-AR) has been identified [15] and characterized in transfected CHO-K1 cells [10]. Given the important role of α_2_-ARs in chromaffin cell physiology with respect to CA secretion regulation [2,7] in the present study we sought to analyze the properties of this polymorphic receptor in PC12 cells, a very well established and widely used chromaffin cell line [13]. In particular, we wanted to see whether the phenotype of the impaired phosphorylation and desensitization of the Del α_2B_-AR also applies in chromaffin cells, and, if so, what consequences this might have on the CA secretion inhibitory (sympatho-inhibitory) function of the receptor in these cells. We found that the agonist-promoted phosphorylation and subsequent desensitization of the Del α_2B_-AR is indeed dramatically impaired in PC12 cells, as well. In particular, it appears that the agonist-induced (i.e. GRK-mediated) phosphorylation and desensitization of the Del α_2B_-AR are deficient, since both of these receptor properties were studied in response to agonist stimulation (UK14304). This is entirely consistent with what has been observed for this receptor variant in another heterologous (but physiologically irrelevant) cell line (COS-7) and in neuronal cells [10,11].

Importantly, in the present study, we report that this impaired phosphorylation and desensitization of the Del α_2B_-AR in PC12 cells leads to enhanced inhibition of Epi and NE secretion by the receptor in this chromaffin cell line. This novel phenotypic/functional finding for this polymorphism is therefore now added to the ever-expanding, over the past several years, list of its (genetic) associations with low basal metabolic rate in some obese populations [15], with acute coronary events [16] and increased risk of sudden cardiac death [17], with impaired endothelial function as assessed by flow-mediated dilatation of the brachial artery [18], with elevated blood pressure in conjunction with stressful work environment [19], with risk reduction for incident diabetes after dietary changes [20], with impaired first-phase insulin secretion that may predict impaired glucose tolerance [21], with reduced autonomic responsiveness by altering cardiac sympathetic and vagal function during sustained handgrip exercise in normotensive obese women [22], and with depressed general autonomic tone and impaired vagal activity in non-diabetic men, which is accentuated by central obesity [23].

Given that α_2_-ARs of the sympathetic nerves and adrenal glands are crucial regulators of SNS activity/outflow and of circulating CA levels in heart failure and in other diseases characterized and aggravated by sympathetic overactivity, by virtue of inhibiting CA secretion and NE release from sympathetic nerve terminals [5], the finding of enhanced inhibition of CA secretion from chromaffin cells by the Del α_2B_-AR reported in the present study is of obvious physiological importance. It strongly implies that carriers of this α_2B_-AR polymorphism might have lower levels of sympathetic outflow, since this polymorphic variant displays enhanced sympatho-inhibitory function due to its impaired desensitization (i.e. termination of signaling). In fact, its previously reported associations with reduced autonomic responsiveness by altering cardiac sympathetic and vagal function and with depressed general autonomic tone and impaired vagal activity [22,23] are entirely consistent with the above postulated effect of this polymorphic α_2B_-AR on sympathetic outflow in vivo.

It is worth noting that whether the α_2B_-AR subtype is endogenously expressed in adrenal chromaffin cells, and in particular in human adrenal chromaffin cells, and participates therein in regulation of CA secretion, is somewhat a matter of debate [5], making the present findings difficult to interpret physiologically. There is however one reported function of α_2B_-AR when expressed in PC12 cells, and that is stimulation of neuronal differentiation of these cells upon chronic epinephrine treatment [24,25]. Thus, in that context, and based on the findings of the present study, this polymorphic Del α_2B_-AR would be expected to stimulate neuronal differentiation of PC12 cells to an even higher extent than its wild-type counterpart.

There appears to be a species-dependent variation in the particular adrenal α_2_-AR subtypes expressed and some contradictory data have been reported in the literature, even for the same species [5]. In fact, our previous study has indicated that only the α_2A _subtype is present endogenously in rat adrenal chromaffin cells (the species that PC12 cells also originate from), and this appears to be the case also in human adrenal glands [7]. Making matters even more complex, PC12 cells do not express any adrenoceptors at appreciable levels endogenously [24,26]. Nevertheless, based on our present findings, it is entirely legitimate to speculate that this polymorphic α_2B_-AR will exert enhanced feedback inhibition of CA release, if present in human adrenal chromaffin cells or wherever in the CNS and in peripheral sympathetic nerve terminals it is expressed in humans in vivo. Therefore, carriers of this α_2B_-AR gene polymorphism might be better protected and experience less severe symptoms from pathological conditions and diseases characterized and aggravated by sympathetic/catecholaminergic overstimulation in vivo, including (but not limited to) heart failure, hypertension, and hyperthyroidism.

## Conclusions

A deletion polymorphism in the human α_2B_-AR gene confers impaired agonist-dependent receptor phosphorylation and desensitization also in the adrenal chromaffin cell line PC12, resulting in enhanced inhibitory function against cholinergic-induced catecholamine secretion in vitro. Thus, this α_2B_-AR gene polymorphism might confer better protection against conditions characterized and aggravated by sympathetic/catecholaminergic overstimulation in vivo, such as heart failure, hypertension, and hyperthyroidism.

## Methods

### Materials

Guanosine diphosphate (GDP), Guanosine 5'-O-(3-thiotriphosphate) (GTPγS), nicotine, epinephrine, 5-bromo-6-(2-imidazoline-2-ylamino)-quinoxaline (UK14304), and oxymetazoline were from Sigma-Aldrich (St. Louis, MO). The anti-phosphoserine (anti-P-Ser) rabbit polyclonal antibody was from Chemicon (Temecula, CA). The anti-α_2B_-AR and anti-GRK2/3 antibodies were from Santa Cruz Biotechnology Inc. (Santa Cruz, CA). [^35^S] Guanosine 5'-O-(3-thiotriphosphate), [^35^S] GTPγS was from Amersham Pharmacia Biotech (Buckinghamshire, UK).

### Cell lines, transfection and cell culture

The cDNA of the human α_2B_-AR (Missouri S&T cDNA Resource Center, Rolla, MO, USA) was modified (Del322-325) using polymerase chain reaction-mediated mutagenesis (Stratagene Cloning Systems, La Jolla, CA). PC12 cells were purchased from American Type Culture Collection (ATCC, Manassas, VA, USA) and transfected with the constructs of interest (i.e. wild type or Del α_2B_-AR) via the Lipofectamine method (Invitrogen, Carlsbad, CA, USA). For comparison of the properties of the two receptors, cell lines with comparable expression levels were chosen, as determined by ligand binding with the α_2_AR-specific antagonist [^3^H]-rauwolscine (3.1 pmol/mg of protein for PC12α_2BWT_, 2.95 pmol/mg of protein for PC12α_2BDel_). The culture of PC12 cells was performed, as described previously [13].

### Receptor phosphorylation studies

After a 10 min stimulation with UK14304 or vehicle PC12 cells were washed three times with ice-cold phosphate-buffered saline and solubilized in 1 ml of a buffer containing 1% Triton X-100, 0.1% SDS, 20 mM Tris-Cl pH 7.5, 125 mM NaCl, 1 mM MgCl_2 _and 1 mM CaCl_2_, protease and phosphatase inhibitors. The α_2B_-ARs were immunoprecipitated with a specific anti-α_2B_-AR antibody and subsequently, the proteins in the supernatant were fractionated on a 12% SDS-polyacrylamide gel, followed by western blotting with an anti-phosphoserine (anti-P-Ser) specific antibody. Equal amounts of protein were loaded in each lane.

### [^35^S] GTPγS binding assay

Cell membranes were prepared by centrifugation and agonist-induced stimulation of [^35^S] GTPγS binding was measured as described previously [14]. Briefly, membrane pellets were resuspended in hypotonic lysis buffer. The reaction was started by adding an aliquot of membrane suspension (15 μg of membrane protein per tube) to reaction buffer (25 mM Tris-HCl, pH 7.4, 5 mM MgCl_2_, 1 mM EDTA, 1 mM dithiothreitol, 100 mM NaCl, 1 μM GDP, and 2 nM [^35^S] GTPγS) with or without agonist (10 μM UK 14304) in a total volume of 100 μl. The incubation was terminated by dilution with 4 volumes of ice-cold 10 mM Tris-HCl buffer, pH 7.4, and vacuum filtration through Whatman GF/B glass fiber filters, which were then placed in scintillation vials for counting in a liquid scintillation counter (Beckman counter). Non-specific binding was measured in the presence of 10 μM GTPγS. For desensitization experiments, cells were pretreated with 10 μM UK 14304 or 10 μM epinephrine for 30 min at 37°C or with vehicle, placed on ice, and washed three times with ice-cold phosphate-buffered saline prior to membrane preparation.

### In vitro catecholamine secretion assay

In vitro epinephrine and norepinephrine secretion in response to various treatments was measured in the supernatant of transfected PC12 cells by ELISA, as described previously [8]. Cells were pretreated with UK14304 (or oxymetazoline) for 30 min prior to the nicotine challenge, and supernatant was collected at 20 min post-nicotine application.

### Statistical analysis

Data are summarized as mean ± SEM. Comparisons were made using *t *tests or ANOVA as appropriate. A Bonferroni correction was applied to the probability values whenever multiple comparisons arose. Values of p < 0.05 were considered significant.

## List of Abbreviations

**α**_2_-AR: α_2_-adrenergic receptor; GPCR: G-protein coupled receptor; GRK: G-protein coupled receptor kinase; KO: knockout; MAPK: Mitogen activated protein kinase; NE: norepinephrine; Epi: epinephrine; PC12: rat pheochromocytoma cell line: [^35^S] GTPγS: [^35^S] Guanosine 5'-O-(3-thiotriphosphate)

## Competing interests

The authors declare that they have no competing interests.

## Authors' contributions

KN carried out the [^35^S] GTPγS binding and in vitro catecholamine secretion assays, and helped to draft the manuscript. TK participated in the receptor phosphorylation experiments. AL conceived of the study, designed it, performed the transfections to establish the PC12 clones and the receptor phosphorylation experiments, and assisted in drafting and editing the manuscript. All authors read and approved the final manuscript.
